# Is Neighborhood Green Space Protective against Associations between Child Asthma, Neighborhood Traffic Volume and Perceived Lack of Area Safety? Multilevel Analysis of 4447 Australian Children

**DOI:** 10.3390/ijerph14050543

**Published:** 2017-05-19

**Authors:** Xiaoqi Feng, Thomas Astell-Burt

**Affiliations:** 1 Population Wellbeing and Environment Research Lab (PowerLab), School of Health and Society, University of Wollongong, Wollongong, NSW 2522, Australia; thomasab@uow.edu.au; 2 Early Start, University of Wollongong, Wollongong, NSW 2522, Australia; 3 Illawarra Health and Medical Research Institute, University of Wollongong, Wollongong, NSW 2522, Australia

**Keywords:** asthma, green space, heavy traffic, air pollution, area safety, Australia

## Abstract

Heavy traffic is a source of air pollution and a safety concern with important public health implications. We investigated whether green space lowers child asthma risk by buffering the effects of heavy traffic and a lack of neighborhood safety. Multilevel models were used to analyze affirmative asthma cases in nationally representative cross-sectional data from 4447 children aged 6–7 years old in Australia. Case-finding was based upon a triangulation of affirmative responses to three questions on doctor-diagnosed asthma, asthma-related medications and illness with wheezing lasting for at least 1 week within the 12 months prior. Among children considered to be exposed to high traffic volumes and areas with 0 to 20% green space quantity, the odds ratio of affirmative asthma was 1.87 (95% CI 1.37 to 2.55). However, the association between heavy traffic and asthma was significantly lower for participants living in areas with over 40% green space coverage (odds ratio for interaction 0.32, 95% CI 0.12 to 0.84). No association between affirmative asthma and green space coverage was observed for participants not exposed to heavy traffic, nor for the area safety variable. Protecting existing and investing in new green space may help to promote child respiratory health through the buffering of traffic-related air pollution.

## 1. Introduction

Field experiments and a rapidly growing number of epidemiological studies indicate potentially important health benefits could be gained from urban greening [1,2,3]. Evidence suggests that general health benefits accrue from different types of green space exposure, including contact with parks and tree canopy [4]. However, evidence on the relationship between green space and child asthma remains mixed. For example a recent cohort study reported children living in greener areas of Vancouver as measured using normalised differential vegetation index (NDVI) had lower odds of incident asthma [5]. In contrast, a study in Sabadell (Spain) found no association between a similar NDVI-based measure of greenness and asthma, though asthma prevalence was reportedly higher with greater proximity to a park [6]. Another study using NDVI to measure greenness was based in Kaunas (Lithuania) and also reported higher odds of child asthma [7]. Finally, a recent longitudinal study in Vancouver reported no association between residential greenness and asthma [8]. Studies of tree canopy and asthma are similarly equivocal. One study in New York City reported lower asthma prevalence in areas with greater tree density [9]. Meanwhile, another study also in New York City reported a higher risk ratio for asthma among children at age 7 living in areas with greater tree canopy coverage [10].

Urban greening could be protective against the development of asthma in children through a range of related pathways. One pathway that has been suggested is that trees and other forms of green space may help to reduce particulate matter and ozone concentrations [11,12,13]. Increased odds of asthma associated with air pollution from traffic and other emissions sources is well known [14,15,16,17,18]. Evidence suggests that experience of a range of psychosocial stressors may also be an effect modifier, potentially intensifying the relationship between asthma and air pollution [19,20,21,22,23]. This effect measure modification may occur through stress enhancing neuro-immune and hypersensitive responses to air pollution [24]. Therefore, the restorative benefits accrued either through views or by being within green spaces may play a buffering role in preventing child asthma over and above the dilution of air pollution concentrations.

Collectively, these hypothesized pathways suggest that the odds of developing asthma among children living in areas with higher air pollution concentrations or proxies thereof (e.g., heavy traffic) may be lower if greater quantities of green space are also present. Conversely, it may be that perceptions of heavy traffic among parents do not necessarily reflect concentrations of air pollution, but perhaps feed more into heightened concerns over general safety for their children that may also influence asthma risk. A lack of neighborhood safety and higher levels of violent crime have been previously shown to be associated with child asthma symptomatology [21,25,26,27,28,29,30]. Accordingly, the purpose of this study was to investigate the potential of green space as an effect modifier of environmental factors associated with asthma risk and to disentangle the extent that traffic volume may be a proxy for area safety, rather than air pollution.

## 2. Methods

### 2.1. Data

Data was extracted from wave 2 of the child cohort collected in 2006 of the Longitudinal Study of Australian Children [31], when the participants were between 6 and 7 years old. A cohort of 4983 participants born between March and February 2000 had been originally recruited in 2004 from the Medicare enrolment database (the most comprehensive data of Australia’s population, affording the opportunity to create a nationally representative sample). The sample was selected in a clustered design stratified across states and territories. There was an average of 40 participants per postcode among the larger Australian states and 20 children per postcode in the smaller states and territories [32]. One child per family was included in the sample and data was collected via face-to-face interviews every two years with their parents and/or guardians (usually the biological mother). Participants’ addresses were linked by the data custodian to the “Statistical Area 2” (SA2) area identifier, developed by the Australian Bureau of Statistics. The “SA2s” are considered to be a surrogate spatial representation for local communities, with approximately 10,000 residents on average [33]. Data from wave 2 was selected to align with the ages of the participants with the International Study of Asthma and Allergies in Childhood [34] and the green space land-use data being measured in 2006 (details below). Of 4464 participants at wave 2, 10 were omitted on the basis of no valid outcome measure and a further seven who did not have an SA2 indicator. These omissions left a final study sample of 4447 participants nested within 1081 SA2s.

### 2.2. Measures

Affirmative asthma was the outcome variable. Case-finding of child asthma at age 6 to 7 years old was based upon a triangulation of affirmative responses to all three of the following questions answered by parents: (i) *“Has a doctor ever told you that [the] study child has asthma?”;* (ii) *“In the last 12 months, has (the) study child taken any medication for asthma?”;* (iii) *“In the last 12 months, has (the) study child had an illness with wheezing in the chest which lasted for a week or more?”* This case-finding approach is aligned with recommendations from the ISAAC study [34] and other studies using the Longitudinal Study of Australian Children [35].

There were three key exposures. The first exposure was parent-reported perceptions of traffic volume within close proximity of the household, serving as a proxy for air pollution concentration. This exposure was ascertained by affirmative responses to the statement: “How strongly do you agree or disagree with these statements about your neighbourhood? There is heavy traffic on my street or road”. Responses ranged from “strongly disagree”, “disagree”, “agree” and “strongly agree.” Area safety was ascertained using similar wording and a range of answers in response to the statement “This is a safe neighbourhood.” The third key exposure was the quantity of green space available as a percentage of land-use within the SA2 of residence. Green space land-use was identified by the Australian Bureau of Statistics “Meshblocks” classified as “parkland” in 2006. Meshblocks classified as “farmland” were not included in the definition of green space quantity as these are not typically areas for public access. The indicator was stratified into 0 to 20%, 20% to 40% and >40% categories.

Participants’ age and gender, maternal highest educational qualification, area socioeconomic disadvantage and geographic remoteness were all taken into account to address potential sources of confounding. Maternal education was ascertained with responses including “secondary school or lower”, “senior secondary school”, “bachelor degree or certificate”, or “graduate diploma or postgraduate qualification.” Area-level socioeconomic disadvantage was measured using the Australian Bureau of Statistics “Socio Economic Index For Areas” (SEIFA) relative index of disadvantage, which takes into account a range of variables including unemployment, educational attainment and income [36].

### 2.3. Statistical Analysis

The patterning of asthma cases with respect to perception of heavy traffic, area safety and green space quantity were examined using cross-tabulations. A further interrogation of sample size and asthma cases across a cross-classification of heavy traffic, area safety and green space quantity was used to explore the potential for effect measure modification. These descriptive analyses were followed up with two-level multilevel logistic regressions with participants at level 1 and SA2s at level 2. Initial modelling tested the odds of affirmative asthma against perceptions of heavy traffic or area safety, adjusting for child age and gender, maternal education, area disadvantage and geographic remoteness. These models were then augmented with the indicator of green space quantity, then with a two-way interaction between green space quantity and perceptions of heavy traffic or area safety to test for potential effect measure modification. Analyses were in MLwIN V2.30 (Centre for Multilevel Modelling, University of Bristol, Bristol, UK) [37], with parameters reported as odds ratios (OR) with 95% confidence intervals (95% CI).

## 3. Results

The percentages of participants living in homes where nearby traffic volume was considered to be high were 20.4%, 19.3% and 21.3% in SA2s (Statistical Area 2) where green space quantity measured 0 to 20%, 20% to 40%, and >40%, respectively. The percentages of participants living in areas considered to be safe were 70.5%, 71.4% and 77.8% were located in SA2s where green space quantity measured 0 to 20%, 20% to 40%, and >40%, respectively. Among those with valid parental responses to the traffic and area safety questions, approximately 26.3% agreed that there was heavy traffic nearby while 94.6% agreed that the area was safe. Cross-tabulations indicated that 25.1% compared to 46.9% agreed that there was heavy traffic among those who agreed and disagreed that the area was safe, respectively. Conversely, 90.2% and 96.1% agreed that the area was safe among those who agreed and disagreed that there was heavy traffic nearby, respectively. The chi-square for this cross-tabulation of perceived area safety and traffic volume was 42.73 (*p* < 0.001).

The prevalence of affirmative asthma in the sample of 4447 participants aged 6 to 7 years old was 8.7% (Table 1). Among the participants living in houses on streets or adjacent to roads considered by parents to have high traffic volumes, the prevalence of affirmative asthma was 11.7%, compared to 7.5% among those where traffic volume was considered low. In SA2s with 0 to 20% green space coverage, the prevalence of affirmative asthma was 9.2%, compared with 8.1% and 7.5% in SA2s with 20% to 40% and >40% green space (chi-square 2.32, *p* = 0.313).

Reasonable cell counts were observed across the cross-classification of green space quantity and perceptions of traffic volume. Among participants whose parents considered local streets to have little traffic, affirmative asthma prevalence varied marginally with respect to green space. Affirmative asthma prevalence was 7.5%, 6.7% and 8.8%, respectively, across SA2s with 0 to 20%, 20% to 40% and >40% green space coverage for participants considered to be exposed to low traffic volumes. For participants considered to be living within the vicinity of heavy traffic volumes, affirmative asthma prevalence was 13.3%, 10.4% and 5.6% across SA2s with 0 to 20%, 20% to 40% and >40% green space coverage, respectively (chi-square 5.65, *p* = 0.059). Asthma prevalence appeared to be lower in SA2s with larger percentages of green space, irrespective of whether participants’ parents felt the area was safe or not safe, albeit these correlations were not statistically significant (safe chi-square 0.86, *p* = 0.652 (not safe chi-square 0.93, *p* = 0.629).

The odds of a case of affirmative asthma were 61% higher among participants living close to high traffic volumes (95% CI 1.25 to 2.09) after adjusting for confounders including area disadvantage and geographic remoteness (Table 2, Model 1). The odds ratio of affirmative asthma relating to traffic volume were virtually unaffected after adjustment for green space quantity as an independent parameter (OR 1.61, 95% CI 1.25 to 2.08). Strata of green space quantity were associated with substantively lower odds of affirmative asthma, though the 95% confidence intervals spanned unity.

An interaction to test for effect measure modification of the odds of affirmative asthma associated with heavy traffic across strata of green space quantity was found to be statistically significant (*p* < 0.05). There was no association between affirmative asthma and green space coverage for participants not exposed to heavy traffic in their place of residence. Among participants considered to be exposed to high traffic volumes and in SA2s with 0**%** to 20% green space quantity, the odds of affirmative asthma were 1.87 (95% CI 1.37 to 2.55). However, the association between heavy traffic and asthma was significantly lower for participants living in areas with over 40% green space coverage (OR for interaction 0.32, 95% CI 0.12 to 0.84).

Multilevel models for the association between affirmative asthma and area safety suggested no statistically significant association between these two variables, although the association was indicative of a protective effect (Table 3). Further adjustment for green space quantity and a two-way interaction between green space quantity and area safety did not reveal any evidence of effect measure modification.

## 4. Discussion

Among a small, but growing evidence base, the balance of prior results appears to lean slightly towards more green space being unfavorable for child asthma [6,7,10]. There are some studies suggesting a potentially protective relationship [5,9] and the analyses reported in this paper adds supportive evidence to that school of thought. The key findings indicate that greater quantities of nearby green space may be protective against child asthma through modification of the effect of heavy traffic volume on streets and roads adjacent to the household of residence. By contrast, area safety was neither associated with elevated asthma risk, nor was any differential association across strata of green space quantity observed. Together, these findings suggest that green space is more likely to be protective of child respiratory health through enhancing local air quality, rather than improving perceived levels of safety in the local community. Finally, the evidence suggests that approximately 40% of an area of residence comprising green space may help to buffer the impacts of heavy traffic exposure on child asthma. This finding suggests the potential for a threshold effect wherein smaller amounts of green space in a neighborhood may have benefits for some health outcomes, but larger quantities may be necessary in order to protect respiratory health.

Heavy traffic volume is a surrogate marker of high air pollution concentrations. There is strong evidence of the types of air pollution referred to as “particulate matter” less than 10 μm in aerodynamic diameter (PM_10_) and less than 2.5 cm (PM_2.5_) being especially dangerous for a range of health outcomes [38,39,40], including child asthma [14,15,17,18]). PM_10_ and PM_2.5_ are not only emitted from vehicle emissions but also from other sources that are likely to be spatially concentrated in areas where traffic volume is heavy, such as tire fragmentation and road dust [41]. If it is practically difficult (or if there is little political appetite) to reduce exposure to these deleterious airborne pathogens in order to help ensure all children have a healthy start in life, the evidence presented in this paper (if reflective of a causal relationship) suggests that protecting existing green spaces and new investments in urban greening may help to partially offset the harms of air pollution on child respiratory health.

A strength of the study was the use of an objective measure of green space quantity, which avoids same-source bias that effects studies using self-reported green space exposures. The use of an internationally recognized means of asthma case finding was another strength, which relied not only upon whether a doctor had made a diagnosis of asthma, but also whether medications for asthma were being taken and an illness involving wheezing had occurred in the past 12 months. This triangulation helps to avoid false positives in the asthma cases. Further strengths were the use of multilevel modelling to analyze a nationally representative source of data spanning a large and topographically diverse country. It is notable that evidence of a potentially protective effect of green space was observed after adjusting for confounders such as geographic remoteness and area disadvantage. These adjustments help to eliminate potential confounding in which apparently protective qualities of green space may be attributable to lower traffic volumes in rural and more advantaged areas. Finally, the analyses of perceived heavy traffic and area safety afforded comparisons of potential pathways for how green space may help to protect child respiratory health.

An important limitation is the potential for exposure misclassification of air pollution concentration. No objective measure of air pollution exposure was available. Instead, this was measured by the proxy indicator of perceived traffic volume. This has been a common approach in the air pollution literature [42,43,44,45,46] due to financial and practical reasons. However, a study suggested that perceived local traffic intensity and objective measures of air pollution are weakly associated among samples of parents in Germany and the Netherlands [47]. Due to the absence of further evidence, it may be that this weak association is due to differences in reference points as to what constitutes high traffic intensity for people living in areas with different topographies. It is unclear as to what extent those findings are subject to under-reporting and potential changes in public understandings of the link between air pollution and traffic intensity through time. It may be that perceptions become more accurate as awareness of potential harms increases and if knowledge of traffic volumes in different areas rises, potentially resulting in more attention paid to traffic volumes (perhaps especially by parents of young children). These are speculations in the absence of further evidence and it is unclear whether any of the aforementioned concerns including the relationship between perceived traffic volume and objective measures of air pollution are generalizable to the Australian context. However, if these concerns are valid, it may be that the true association is stronger than reported in this paper.

This is among the first studies to combine the research on area safety and asthma with green space and asthma. There have been studies prior to this that have examined associations between green space and perceived safety (e.g., [48]), but rarely in the context of childhood asthma. The lack of a significant association for area safety and asthma in this paper was surprising, given prior theorizing on the impact of psychosocial stress [28] and previous studies not only of area safety [26,27], but also related contextual factors such as neighborhood violence [29,49]. In this study, very large percentages of participants lived in areas considered by their parents to be safe, which is higher than in several of the aforementioned studies. It may be that the factors which determine perceptions of area safety are not universal and patterned differently across national boundaries and cultural contexts. It may also be that it is not the perceptions of safety held by the parent that are most important, but those harbored by the child. As such, this paper does not rule out the potential role of area safety as a determinant of asthma nor the potential for green space to be an effect modifier of that association, despite the null findings. Importantly, unlike for traffic volume where the amount of green space may be more salient, for the association between asthma and area safety the design and features within green spaces may also be important as they have been shown to be related to indicators of stress and mental wellbeing [50,51,52].

No evidence was found in this paper to support prior reports of unfavorable association between green space and asthma [6,7,10]. However, those reports are not unfounded, given that asthma might be triggered by production of pollen [53] and exposure to pesticides [54] that may be more common in areas with more green space. There have been reports, for example, of area greenness associated with allergic rhinitis and eye and nose symptoms in urban contexts but the opposite association in rural environs [55]. This points to the potential complexity that future studies of green space and health may need to embrace. Analyses of different types of vegetation located within each SA2 were not available in this paper, but this would seem to be an important avenue for testing new hypotheses on the relationship between greenness and allergies.

The findings presented in this paper also raise several other opportunities for future research in order to take this field of enquiry forwards. The first is to link objective indicators of air pollution to support or falsify the results presented in this paper of effect measure modification of asthma and perceived traffic volume across strata of green space quantity. Second, similar studies of effect modification via objectively measured and perceived markers of air pollution exposures ought to be conducted in other parts of the world to ascertain if the protective qualities of green space for child respiratory health are universal or if responder bias plays much of a role in different contexts. Third, the utility of green space quantity and quality as effect modifiers of a potential association between asthma and neighborhood safety also requires more attention. Fourth, other studies of effect measure modification are required and, where possible, need to incorporate exploration of threshold effects that could be translated into urban planning guidelines and policy. Finally, the study is cross-sectional and it would be pertinent for longitudinal studies to be conducted to examine the degree of protection conveyed by green spaces to respiratory health across childhood and beyond.

## 5. Conclusions

As green spaces are increasingly viewed as a resource for preventive health and advocates lobby for the protection of existing green spaces and investment in new ones on the basis of health benefits, it is essential that epidemiological studies keep pushing to identify when, where, for whom and how much green space is needed in every neighborhood to give all children the healthiest start in life. The findings from this study enhance existing evidence that suggests a potential buffering effect of green space against air pollution on birth outcomes by reporting a reduced risk of affirmative asthma among children living in greener areas, despite heavy traffic nearby.

## Figures and Tables

**Table 1:** Cross-tabulation of the study sample and prevalence of affirmative asthma with respect to green space quantity and parental perception of heavy traffic.

Green Space Quantity	0% to 20%	20% to 40%	>40%	Total
N (% Affirmative Asthma)				
Heavy Traffic				
Disagree	1584 (7.5%)	625 (6.7%)	308 (8.8%)	2517 (7.5%)
Agree	581 (13.3%)	211 (10.4%)	108 (5.6%)	900 (11.7%)
No response	681 (9.5%)	257 (9.3%)	92 (5.4%)	1030 (9.1%)
Safe Area				
Disagree	122 (12.3%)	42 (7.1%)	19 (10.5%)	183 (10.9%)
Agree	2007 (8.7%)	780 (7.7%)	395 (7.8%)	3182 (8.4%)
No response	717 (9.9%)	271 (9.2%)	94 (5.3%)	1082 (9.3%)
Total	2846 (9.2%)	1093 (8.1%)	508 (7.5%)	4447 (8.7%)

**Table 2:** Associations and effect measure modification of the association between heavy traffic and child affirmative asthma across strata of green space quantity.

Variables	Model 1	Model 2	Model 3
Odds Ratio (95% Confidence Interval)			
Heavy Traffic (Ref: Disagree)			
Agree	**1.61 (1.25, 2.09)**	**1.61 (1.25, 2.08)**	**1.87 (1.37, 2.55)**
Green Space Quantity (Ref: 0**%** to 20%)			
20% to 40%		0.87 (0.66, 1.15)	0.89 (0.61, 1.30)
>40%		0.79 (0.54, 1.15)	1.15 (0.73, 1.82)
Heavy Traffic *x* Green Space Quantity			
Agree *x* 20% to 40%			0.84 (0.45, 1.58)
Agree *x* > 40%			**0.32 (0.12, 0.84)**

All models adjusted for age (months), gender, maternal education, household income, geographic remoteness and area disadvantage; **bold**: *p* < 0.05.

**Table 3:** Associations and effect measure modification of the association between heavy traffic and child affirmative asthma across strata of green space quantity.

Variables	Model 1	Model 2	Model 3
Odds Ratio (95% Confidence Interval)			
Safe Area (Ref: Disagree)			
Agree	0.73 (0.45, 1.20)	0.73 (0.45, 1.20)	0.67 (0.38, 1.19)
Green Space Quantity (Ref: 0**%** to 20%)			
20% to 40%		0.87 (0.66, 1.15)	0.54 (0.14, 2.06)
>40%		0.79 (0.54, 1.16)	0.85 (0.17, 4.17)
Safe Area *x* Green Space Quantity			
Agree *x* 20% to 40%			1.60 (0.41, 6.27)
Agree *x* > 40%			1.01 (0.20, 5.21)

All models adjusted for age (months), gender, maternal education, household income, geographic remoteness and area disadvantage.
